# Enhanced localization of anticancer drug in tumor tissue using polyethylenimine-conjugated cationic liposomes

**DOI:** 10.1186/1556-276X-9-209

**Published:** 2014-05-05

**Authors:** Hee Dong Han, Yeongseon Byeon, Hat Nim Jeon, Byung Cheol Shin

**Affiliations:** 1 Department of Immunology, School of Medicine, Konkuk University, 268 Chungwondaero, Chungjusi, Chungcheongbukdo 380-701, South Korea; 2 Research Center for Medicinal Chemistry, Division of Drug Discovery Research, Korea Research Institute of Chemical Technology, 141 Gajeong-ro, Yuseong-gu, Daejeon 305-600, South Korea

**Keywords:** Liposome, Polyethylenimine, Tumor, Localization

## Abstract

Liposome-based drug delivery systems hold great potential for cancer therapy. However, to enhance the localization of payloads, an efficient method of systemic delivery of liposomes to tumor tissues is required. In this study, we developed cationic liposomes composed of polyethylenimine (PEI)-conjugated distearoylglycerophosphoethanolamine (DSPE) as an enhanced local drug delivery system. The particle size of DSPE-PEI liposomes was 130 ± 10 nm and the zeta potential of liposomes was increased from -25 to 30 mV by the incorporation of cationic PEI onto the liposomal membrane. Intracellular uptake of DSPE-PEI liposomes by tumor cells was 14-fold higher than that of DSPE liposomes. After intratumoral injection of liposomes into tumor-bearing mice, DSPE-PEI liposomes showed higher and sustained localization in tumor tissue compared to DSPE liposomes. Taken together, our findings suggest that DSPE-PEI liposomes have the potential to be used as effective drug carriers for enhanced intracellular uptake and localization of anticancer drugs in tumor tissue through intratumoral injection.

## Background

Liposome-based approaches, which show great potential for cancer therapy, allow for the development of a broad armamentarium of targeted drugs [1-3]. However, one of the key challenges in the application of liposomal drug delivery for chemotherapy is the requirement of efficient drug localization in tumor tissue. These liposomal systems are normally injected intravenously for systemic application. The effectiveness of intravenously delivered liposomes, however, is plagued by problems such as rapid opsonization and uptake by the reticuloendothelial system (RES), resulting in inefficient delivery [4-6]. Therefore, novel delivery systems to overcome such limitations are thus in urgent need.

Under localized conditions, drug delivery systems formulated to deliver high concentration of drugs over an extended period could be an ideal strategy to maximize the therapeutic benefit and avoid possible side effects [7]. However, because low molecular weight drugs can rapidly pass into the bloodstream after intratumoral injection and because the retention time of such drugs in tumors is considerably short, new strategies to enhance the drug delivery and therapeutic effects in tumor tissues are needed.

In this study, we present a novel method for drug delivery using polyethylenimine (PEI)-incorporated cationic liposomes, which can be injected directly into the tumor site. PEI is a synthetic cationic polymer that has been extensively used to deliver oligonucleotides, siRNA, and plasmid DNA *in vitro* and *in vivo*[8-10]. Moreover, the cationic charge of the carrier surface can be enhanced through the intracellular uptake of vehicles to negatively charged tumor cells or tissues [11-13]. After penetration of cationic PEI liposomes into the cells, PEI has a protonatable nitrogen atom, which enables the ‘proton sponge’ effect over a wide range of pHs in the endosome. Consequently, PEI buffers acidification within the endosome after endocytosis, resulting in osmotic swelling and cell rupture allowing for endosomal escape of the PEI/siRNA polyplexes [14]. Although cationic PEI has promising potential as a vehicle, it also presents some of the toxicity problems associated with other non-viral vectors [15,16]. PEI can, however, be modified for reduced toxicity, and its free amine groups can be used to conjugate cell binding or targeting ligands [17-19]. Therefore, we selected PEI to increase localization of liposomes in tumor micro-environment in this study.

Cationic liposomes can also be simply injected at the target site without the need for surgical procedures. The PEI-incorporated cationic liposomes system, thus, has the potential to enhance the concentrations of therapeutic payloads at the tumor site, minimize possible side effects, and ultimately increase the therapeutic index of therapies. Although many cancers metastasize, several types of external cancers such as skin, breast, or neck cancer may be amenable to treatment using DSPE-PEI liposomes. Here, we demonstrate that the anticancer drug delivery system based on cationic liposomes is potentially a novel and powerful local drug delivery system for therapeutic agents.

## Methods

### Materials

Polyethylenimine (PEI, MW, 600 g/mol), glutaric anhydride (GA), 1-[3-(dimethylamino) propyl]-3-ethylcarbodiimide hydrochloride (EDC), and *N*-hydroxy-succinimide (NHS) were purchased from Sigma Aldrich Co. (Milwaukee, WI, USA). Chemicals 1,2-distearoyl-*sn*-glycero-3-phosphoethanolamine (DSPE), l-α-phosphatidylcholine (soy-hydrogenated) (HSPC), and cholesterol (CHOL) were purchased from Avanti Polar Lipids Inc. (Alabaster, AL, USA). The anticancer drug doxorubicin (DOX) was obtained from Boryung Pharm. Co. (Ansan, Korea) and calcein was purchased from Sigma Co. (St. Louis, MO, USA). All other materials and solvents were of analytical grade and used without further purification.

### Synthesis of DSPE-PEI

**Figure 1:**
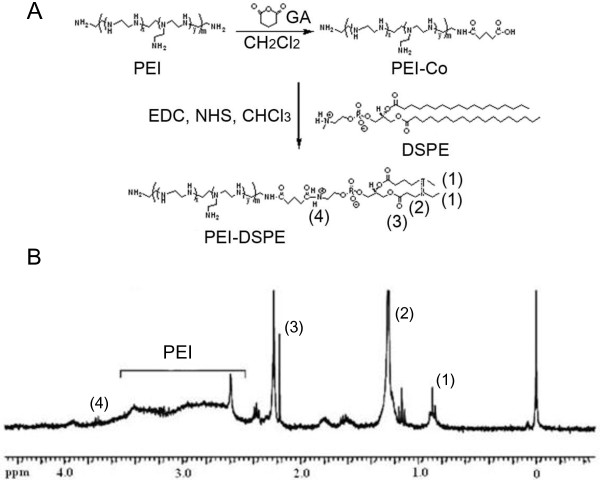
**Chemical structure (A) and **^**1**^**H-NMR spectra (B) of synthesized DSPE-PEI.**

DSPE-PEI conjugate was synthesized according to methods described in our previous study with a minor modification [20]. To prepare carboxylated PEI (PEI-co), 1 mmol of PEI (MW 10 kDa) was dissolved in 50 ml of methylene chloride (MC) solution, which was then added to 0.1 mmol of GA (dissolved in 10 ml of MC) solution, followed by refluxing at room temperature for 10 h. MC was then removed using a rotary evaporator at 20°C to produce carboxylated PEI-co (Figure 1A). To synthesize carboxylated PEI-co and DSPE, PEI-co (0.5 g, 0.83 mmol) and EDC (0.83 mmol) were treated with NHS (0.83 mmol) in 50 ml of chloroform at 27°C for 30 min. DSPE (0.83 mmol) was dissolved in 20 ml of chloroform and then mixed with the PEI-co solution for conjugation of DSPE via amide linkage at 27°C for 10 h. After synthesis of DSPE-PEI, the residual chloroform was removed by rotary evaporator at 20°C. Following synthesis, DSPE-PEI was purified via dialysis for 2 days at 4°C using cellulose dialysis tubing (MWCO 12000, Viskase Co., Darien, IL, USA). DSPE-PEI powder was obtained through a lyophilization process using a freeze-dryer (Ilshin Lab Co., Korea) and stored at 4°C until use. The chemical structure of synthesized DSPE-PEI is shown in Figure 1A.

### Preparation of liposomes

DOX-loaded cationic liposomes were prepared using the remote loading method by employing ammonium sulfate gradient [21,22]. Lipid compositions of the prepared control (DSPE) and DSPE-PEI liposomes were HSPC/CHOL (4 mg of lipid) and HSPC/CHOL/DSPE-PEI (0.1 mg, 0.4 mg, 0.7 mg, and 1 mg of DSPE-PEI based on HSPC/CHOL formulation), respectively. Lipids were dissolved in chloroform, dried on a thin film on a rotary evaporator (Buchi Rotavapor R-200, Switzerland), and finally suspended in a 250 mM of ammonium sulfate solution. The liposomal solution was extruded by passing it through a polycarbonate filter (pore size, 100 nm, Whatman, Piscataway, NJ, USA) using an extruder (Northern Lipids Inc., Burnaby, Canada). Free ammonium sulfate was removed by dialysis for 48 h at 4°C using cellulose dialysis tubing (MWCO 3500, Viskase Co., Darien, USA). The liposomal solution was mixed with a 2 mg/ml DOX solution and incubated for 2 h at 60°C after which the mixture was dialyzed to facilitate the removal of free DOX. DOX-loaded liposomes were stored at 4°C until use. In addition, to DOX-loaded liposomes, calcein-loaded liposomes were prepared for assessment of the localization in tumor-bearing mice. Calcein-loaded liposomes with the above-mentioned compositions were prepared by loading calcein serving as a model drug in liposomes using the pH gradient method [23].

The particle size and zeta potential of liposomes were measured by laser light scattering using a particle size analyzer (ELS-8000, Outskate, Seongnam, South Korea). The loading efficiency of DOX into liposomes was measured by fluorescence spectrophotometry (Barnstead, Apogent Tech., Dubuque, IA, USA) at excitation and emission wavelengths of 490 and 590 nm, respectively.

### Cell line and mice

The human lung carcinoma cell line A549 was cultured in RPMI 1640 medium supplemented with 10% fetal bovine serum (FBS), 50 units/ml penicillin-streptomycin, 2 mM l-glutamine, 1 mM sodium pyruvate, 2 mM non-essential amino acids, and 0.4 mg/ml G418. The cell culture was sustained at 37°C in a 5% CO_2_ incubator, and the cells were maintained in the exponential growth phase. Male BALB/c *nu/nu* nude mice (5 weeks old, 20 to 22 g) were purchased from Japan SLC Inc. (Hamamatsu, Shizuoka, Japan). All procedures involving animals were performed according to approved protocols and in accordance with the recommendations specified in the NIH guidelines for proper use and care of laboratory animals.

### Flow cytometry analysis

A549 cells were plated in a 6-well plate at a density of 2 × 10^5^ cells per well and cultured in medium supplemented with 10% FBS and 1% penicillin (Life Technologies, Carlsbad, CA, USA) at 37°C. Culture medium was replaced with 2 ml per well of culture medium containing liposomal solutions (30 μg DOX/ml). The cells were incubated with liposomes for 2 h at 37°C in a 5% CO2 incubator. After incubation, the cells were washed three times with phosphate-buffered saline (PBS). The intracellular uptake efficiency of liposomes by A549 cells was monitored by flow cytometry (FACScan, Becton Dickinson, Franklin Lakes, NJ, USA) using CELLQuest software (Becton Dickinson Immunocytometry System, Mountain View, CA, USA), and the morphology of tumor cells containing DOX-loaded liposomes was observed by fluorescence microscopy (Olympus CKX 41, Shinjuku-ku, Tokyo, Japan).

### Cytotoxicity test

The cytotoxicity of liposomes in A549 cells was determined by MTT assay. A549 cells were seeded into 96-well plates at a density of 1 × 10^3^ cells per well and cultured in liposomal solution containing culture medium 37°C for a predetermined time. The absorbance was measured at 590 nm using a microplate reader (EL808, Bio-Tek, Instruments, Winooski, VT, USA).

### Localization of DSPE-PEI liposomes in tumor tissue

A549 (1 × 10^6^) cells were subcutaneously injected into BALB/c *nu/nu* nude mice. Four weeks after injection, free calcein was used as a model drug or liposomal calcein was injected intratumorally into the mice, after which the tumor tissue was monitored continuously for 4 h. The localization efficiency of liposomes in tumor tissues of the live tumor-bearing mice was directly observed under a fluorescence microscope (Macro-Imaging System Plus LT-9macimstsplus, Lightools Research, Encinitas, CA, USA) equipped with Image-Pro Plus software (Media Cybernetics, Silver Spring, MD, USA).

## Results and discussion

### DSPE-PEI synthesis

The synthesis of DSPE-PEI conjugate was confirmed by proton NMR analysis. Figure 1 shows the chemical structures and ^1^H-NMR spectra of the synthesized DSPE-PEI conjugate. As shown in Figure 1B, peaks corresponding to the CH_3_ (1) and CH_2_ (2,3, and 4) protons were observed at 0.8 to 1.0 ppm (1), 1.1 to 1.4 ppm (2), 2.1 to 2.3 ppm (3), and 3.7 to 3.8 ppm (4), respectively. In addition, the PEI peaks were observed at 2.5 to 3.5 ppm. The synthesis yield was approximately 93%.

### Characteristics of liposomes

**Figure 2:**
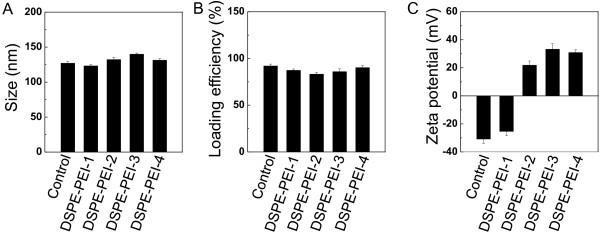
**Physical properties of liposomes.** Liposome size **(A)**, loading efficiency of DOX **(B)**, and zeta potential of the liposomal surface **(C)**. Control represents DSPE liposomes. PEI-1, PEI-2, PEI-3, and PEI-4 represent PEI contents of 10%, 40%, 70%, and 100% (*w*/*w* total lipid) in liposomal formulations, respectively. Data shown represent means ± SD (*n* = 3).

The physical properties of DSPE-PEI liposomes are shown in Figure 2. The mean particle size of DSPE-PEI liposomes was approximately 120 to 140 nm, and the loading efficiency of DOX was 90% to 93% (Figure 2A,B). The particle size and loading efficiency of liposomal formulations did not show significant difference. Particle size is an important factor for penetration of liposomes into cells or organs [24]. Raasmaja et al. reported that smaller (MW 22 kDa) linear PEI-attached liposomes were more effective in gene transfection than larger (MW 25 kDa) branched PEI-attached liposomes and that particle size also decreased when linear PEI polymer was used instead of branched PEI [25]. In this study, small (MW 10 kDa) linear PEI polymers were used and therefore, the PEI concentration on the liposomal surface may not affect the particles size. DSPE-PEI liposomes were found to be uniform in size and small enough for efficient tissue and cell penetration. The zeta potential of DSPE-PEI liposomes changed from -35 to 30 mV with the addition of PEI (Figure 2C), demonstrating that the addition of the cationic lipid onto the liposomal surface induced a positive surface charge on the liposomes. A PEI content of as much as 0.4 mg, however, resulted in a leveled off surface charge, indicating that the surface of the liposomes may have been saturated at a PEI concentration of 0.4 mg. Positively charged vehicles exhibit enhanced intracellular delivery via an electro-binding effect between the positive liposomal surface and negative cell surface [11] and therefore, surface charge is also an important factor in the efficacy of intracellular delivery of liposomes.

### Intracellular delivery of DSPE-PEI liposomes

**Figure 3:**
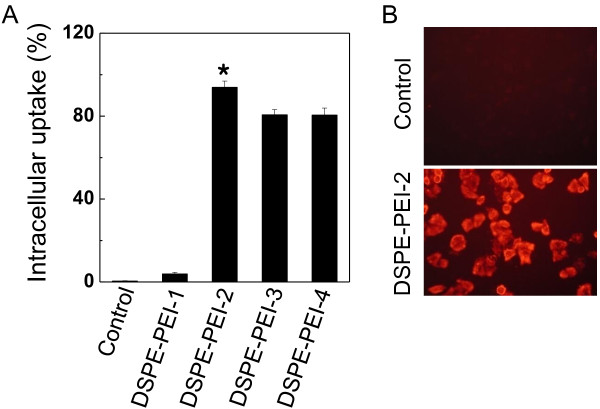
**Intracellular uptake of liposomes. (A)** Flow cytometric assessment of the intracellular delivery of liposomes to tumor cells. **(B)** Assessment of the intracellular uptake of liposomes by A549 tumor cells using fluorescence microscopy. PEI-1, PEI-2, PEI-3, and PEI-4 represent PEI contents of 10%, 40%, 70%, and 100% (*w*/*w* total lipid) in liposomal formulations, respectively. Error bar represents mean ± SD (*n* = 3); *p < 0.001.

Next, the intracellular uptake of liposomes with different surface charges was assessed. The intracellular uptake was measured and monitored using flow cytometry and fluorescence microscopy, respectively (Figure 3). While control (DSPE) liposomes exhibited low intracellular delivery efficiency (0.5%) because of the negatively charged liposomal surface, DSPE-PEIs exhibited increased intracellular efficiency (up to 80%) compared to control liposomes. Notably, the intracellular uptake of DSPE-PEI-2 liposomes was significantly higher than that of control liposomes (*p* < 0.01, Figure 3A). These findings indicate that an effective attachment took place between the cationic DSPE-PEI liposomes and the negatively charged cell surface and that the intracellular uptake of liposomes was enhanced by the electric interaction of liposomes with tumor cells [11,25]. Based on these results, DSPE-PEI-2 (0.4 mg of DSPE-PEI) liposomes were selected for further study. In addition, we check the intracellular uptake of liposomes in tumor cell by fluorescence microscopy (Figure 3B). The uptake of DSPE-PEI-2 liposomes by tumor cells was considerably higher than that of control liposomes. This result further supports our hypothesis by demonstrating an electric interaction between a negatively charged tumor cell surface and positively charged DSPE-PEI-2 liposomes.

### Cytotoxicity assay

**Figure 4:**
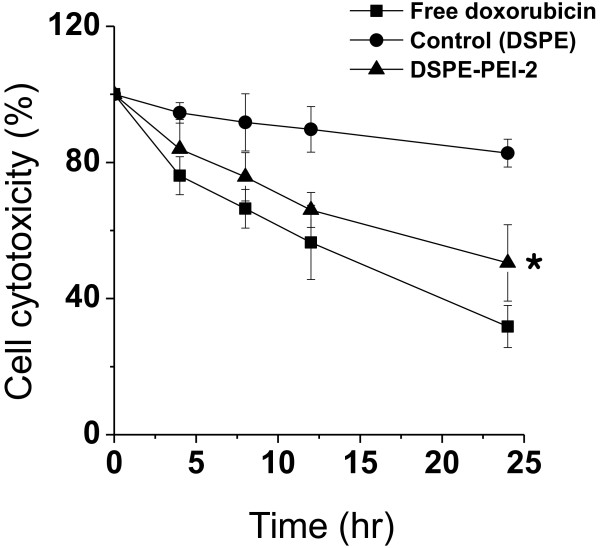
**Cytotoxicity after liposomal DOX uptake in A549 cells.** Error bar represents mean ± SD (*n* = 3); *p < 0.05.

Prior to assessing the *in vivo* localization of DSPE-PEI-2 liposomes, the *in vitro* cytotoxicity of free DOX (positive control), control liposomes (negative control), and DSPE-PEI-2 liposomes was measured in A549 cells using an MTT assay (Figure 4). Free DOX was found to be more cytotoxic to A549 cells than liposomal DOX due to the higher cellular uptake of free DOX by tumor cells via diffusion mechanisms [26,27]. Furthermore, DSPE-PEI-2 (cationic liposomes) also showed significantly higher cytotoxicity compared to control liposomes (*p* < 0.01). The lower cytotoxicity of control liposomes may be a result of their low intracellular uptake. Cellular uptake of negatively charged control liposomes was inhibited as demonstrated by the measured zeta potential (Figure 2C) and by the flow cytometric study (Figure 3A). DSPE-PEI-2 liposomes, on the other hand, do interact electrostatically with A549 cell membranes, resulting in increased cytotoxicity of DOX-loaded DSPE-PEI liposomes.

### Tumor tissue localization of liposomes

**Figure 5:**
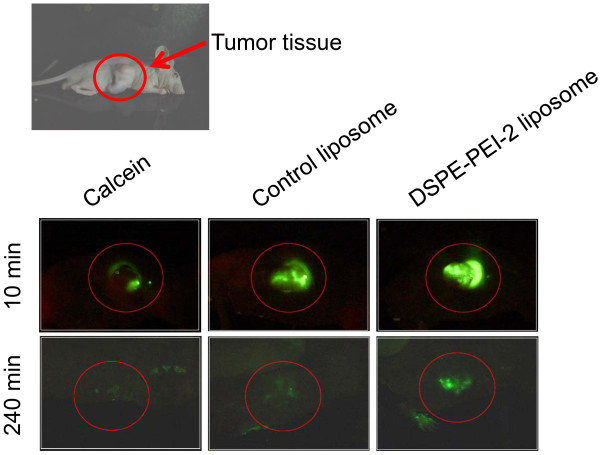
**Fluorescence microscopy images of liposomal calcein in A549 tumor-bearing mice following intratumoral injection.** Localization of liposomes in the tumor tissue was directly observed by fluorescence microscopy in live tumor-bearing mice.

The possible role of cationic charge in enhancing the accumulation of liposomes in tumor tissue was assessed by fluorescence microscopy. Figure 5 shows the localization of free calcein, control liposomes (negative charge), and DSPE-PEI-2 liposomes (positive charge) in tumor-bearing mice after intratumoral injection. As shown in Figure 5, the image of DSPE-PEI-2 liposomes exhibits prominent fluorescence 10 min after injection, and DSPE-PEI-2 liposomes at the tumor site show a longer retention time (240 min) than either control liposomes or free calcein. This result implies that the interaction of tumor vessels with cationic liposomes, specifically with DSPE-PEI-2 liposomes, may occur electrostatically between the negative cell surfaces and positive DSPE-PEI-2 liposomes. The observed effect is likely a result of the surface charge of the cationic liposomes that were not taken up by the tumor tissue, resulting in an enhancement of the localization efficiency of the cationic liposomes. Toward increasing the localization of payloads, extensive research investigation has been carried out into methods of modifying various carriers including ligand-labeled liposomes [28], hydrogel-based intratumoral injections [7], and magnetic-based carriers [29]. Although these investigations have yielded promising results, the additional formulations of such carrier systems require optimization.

## Conclusions

Intratumoral injection is an effective method for liposome-mediated drug delivery into tumor tissues. The use of DOX-loaded DSPE-PEI cationic liposomes was found to result in significantly increased *in vitro* intracellular uptake compared with control liposomes. Notably, the conjugation of PEI to the liposomal membrane effectively improved the localization of drug-loaded liposomes at the tumor site through electrostatic interaction, which occurred in the tumor tissue of tumor-bearing mice treated with intratumorally injected liposomes. Our results demonstrate a promising approach to improve the intracellular uptake and localization effect of cationic liposomes. Although DSPE-PEI liposomes exhibit enhanced intracellular uptake, additional studies on the localization, injection route, and stability of these carriers is required for validation of their potential clinical application. The cationic liposome delivery strategy presented here has considerable potential as a drug delivery platform for the treatment of a broad range of human diseases and can be adapted for other injection applications in various therapeutic fields.

## Competing interests

The authors declare that they have no competing interests.

## Authors’ contributions

YB performed the preparation and characterization of the liposomes. HNJ participated in the intracellular uptake and cell cytotoxicity assay. HDH and BCS conceived of the study and participated in its design and coordination. All authors read and approved the final manuscript.
